# Investigation of Depression, Anxiety, Sleep Quality, and Fatigue in Thalassemia Major Patients: A Study of the Correlation Between Sleep Quality and Laboratory Findings

**DOI:** 10.7759/cureus.72614

**Published:** 2024-10-29

**Authors:** Deniz Sener, Sevil Sadri, Vildan Gursoy

**Affiliations:** 1 Department of Medicine, Bursa City Hospital, Bursa, TUR; 2 Department of Hematology, Bursa City Hospital, Bursa, TUR

**Keywords:** beta-globin, chronic illness, fatigue, sleep quality, thalassemia major

## Abstract

Introduction: Thalassemia major (TM) experiences a high rate of psychiatric problems, with frequent occurrences of depression and anxiety as well as a high incidence of insomnia. In the present study, we aimed to investigate the severity of depression, anxiety, sleep disorders, sleep quality, and fatigue and explore the relationship between sleep quality and laboratory parameters.

Methods: Twenty-eight patients and 37 healthy volunteers were included. Pittsburgh Sleep Quality Index (PSQI), Insomnia Severity Index (ISI), Epworth Sleepiness Scale (ESS), Beck Depression Inventory, Beck Anxiety Inventory, and Fatigue Severity Scale were used.

Results: We found 46.43% of TM patients had depression, 78.5% had mild anxiety, and 3.57% had severe anxiety. ISI score (p = 0.027), ISI subthreshold insomnia percentage (p = 0.027), PSQI global score (p = 0.002), PSQI insomnia percentage (p = 0.025), PSQI subjective sleep quality (p = 0.008), PSQI sleep latency (p = 0.003), fatigue score (p = 0.026), and chronic fatigue (p = 0.037) percentage were significantly higher in the patients group than in the controls group. Ferritin was positively correlated with PSQI subjective sleep quality (r = 0.478, p = 0.010) and fatigue score (r = 0.427, p = 0.023). Heart involvement in magnetic resonance imaging was positively correlated with ISI score (r = 0.426, p = 0.024) and ISI classification (r = 0.455, p = 0.015).

Discussion: Depression and anxiety were significantly higher in TM patients compared to healthy controls. Chronic fatigue and insomnia were more common in TM patients, with subjective sleep quality being impaired and sleep latency prolonged. Furthermore, it was observed that individuals with higher ferritin levels had higher scores of subjective sleep quality and fatigue. Additionally, as cardiac involvement increased, severity of insomnia also increased.

## Introduction

Thalassemia major (TM) is a homozygous autosomal recessive disorder caused by a deficiency in beta-globin chain synthesis. TM is the cause of chronic hemolytic anemia, and patients often require blood transfusions from early childhood. The disease is characterized by transfusion-dependent anemia, ineffective erythropoiesis, and iron deposition in organs [1,2]. Studies have reported that 3%-10% of the world population carries the thalassemia gene and 60,000 thalassemic babies are born annually [3,4]. The prevalence of TM is higher in the Mediterranean region, Middle East, and Southeast Asia, with a significant number of patients in Turkey [5].

Accumulation of excessive iron in organs due to blood transfusions and chelation therapy in patients with TM leads to cardiac and glandular involvement, resulting in the premature death of patients at a young age [6]. Implementing proper transfusion regimens and effectively managing complications related to chelation therapy have enabled an extension of patients' lifespans and improved their quality of life [7]. Physical changes such as bone deformities, short stature, and distinctive facial features resulting from ineffective erythropoiesis cause patients to feel different from healthy people. Frequent hospital stays brought on by chronic illnesses, transfusions, chelation therapy, and endocrine gland involvement that causes diabetes, infertility, and sexual issues frequently cause patients to face social obstacles like finding work and getting married [8-10]. Numerous studies have demonstrated that TM patients experience a high rate of psychiatric problems, with frequent occurrences of depression and anxiety as well as a high incidence of insomnia [11-14].

Among all mental illnesses, generalized anxiety disorder most likely shares the most similarities with the etiology of sleeplessness. More than half of patients with generalized anxiety disorder are reported to have insomnia, and anxiety disorders are frequently linked to insomnia among psychiatric diseases. According to the hypothalamus-pituitary-adrenal system theory and the hyperarousal hypothesis, anxiety disorders and insomnia are believed to have a shared pathophysiology [15-17]. Although depression is thought to be a continuum condition that includes anxiety disorders and insomnia, polysomnography (PSG) results in depressed patients have revealed traits like increased rapid eye movement (REM) density and shortened REM sleep latency, in addition to findings that are typical of insomniacs and patients with generalized anxiety disorder, like decreased [18].

Additionally, due to the infective erythropoiesis seen in TM patients, the nasal root is compressed, and the forehead and facial bones protrude, resulting in a distinctive facial appearance. This can lead to disturbances in sleep quality, daytime sleepiness, and reduced performance in daily activities [19].

Sleep is considered one of the fundamental physiological needs of the human body and the most vital human needs. Sleep enables us to continue daily activities and provides the necessary energy for our mental and physical health. Quality sleep helps maintain our quality of life. However, physical and mental illnesses can disrupt a person's life and sleep quality, and insomnia can also lead to physical problems, such as fatigue, cardiovascular diseases, hypertension, and diabetes, and mental problems, such as neurocognitive impairment, processing speed, switching attention, and several short-term visual memory errors. Sleep-related issues can cause changes in appetite, lack of concentration, depression, mood swings, and fatigue. Anxiety, depression, and fatigue reduce quality of life [20]. Increased daytime sleepiness due to fatigue, physical reasons, and fluctuating mood affects sleep quality.

It is important to investigate and diagnose problems, such as quality of life, depression, education status, and long-term planning uncertainties about the future, related to various aspects of life during follow-ups of TM patients to improve their quality of life through proper treatment. Previous studies in TM patients have explored the relationship between sleep quality and blood parameters [21]. In the present study, we aimed to investigate the severity of depression, anxiety, sleep disorders, sleep quality, and fatigue that could be attributed to physical and social reasons in TM patients and the relationship between sleep quality and laboratory parameters.

## Materials and methods

This study included 28 patients (20 women and eight men) aged 18 years and above under follow-up due to beta-TM at the Thalassemia Center of Bursa City Hospital, Bursa, Turkey, between May 2023 and February 2024. Additionally, 37 healthy volunteers (28 women and nine men) aged 19-35 years were included as controls. All patients who agreed to participate in the study and who met the inclusion criteria with follow-up at our hospital's thalassemia center were included. Hemoglobin levels, chelation therapy use, splenectomy presence, annual number of blood transfusions, and liver and cardiac magnetic resonance imaging (MRI) findings of the patients were recorded. Cardiac MRI (T2*) is a sensitive method that shows iron accumulation in the heart. Liver MRI-T2 is an effective method that indirectly measures liver iron accumulation. Since it is a noninvasive method that can be performed in many centers, measuring it once a year or when the treatment is changed is recommended. The aim is to start measuring patients over the age of 10 in applicable centers and keep the level above 20 ms with chelation treatments. Patients and healthy controls were assessed using the Pittsburgh Sleep Quality Index (PSQI), Insomnia Severity Index (ISI), Epworth Sleepiness Scale (ESS), Beck Depression Inventory (BDI), Beck Anxiety Inventory (BAI), and Fatigue Severity Scale (FSS) through face-to-face interviews conducted by a same neurologist. Healthy volunteer controls were selected from individuals without chronic diseases or neurological complaints who had unremarkable neurological examinations. The study included patients and healthy controls without medical conditions affecting sleep quality, without the use of antidepressants or sleeping pills, and without a habit of substance abuse. The PSQI consists of 10 questions covering the past month. The questions are divided into seven subscales, including subjective sleep quality, sleep latency, sleep duration, sleep efficiency, sleep disturbances, use of sleep medication, and daytime dysfunction. Each group is scored between 0 and 3 points. The total PSQI score is obtained by adding the subscale scores. In the present study, a PSQI score of 5 and above was considered poor sleep quality. The ESS consists of eight questions, each scored on a scale of 0-3. A total score of 0-5 points was considered normal, 6-10 points indicated normal but increased daytime sleepiness, 11-12 points indicated increased but mild daytime sleepiness, 12-15 points indicated increased and moderate daytime sleepiness, and 16-24 points indicated increased and severe daytime sleepiness. ISI consists of five questions, with the first question having three subitems. Each was scored from 0 to 4; a total score of 0-7 was considered clinically insignificant insomnia, 8-14 points indicated subthreshold insomnia, 15-21 points indicated moderate insomnia, and 22-28 points indicated severe insomnia. In the present study, a BDI score of ≥17 points was considered depression. BAI scores ranged between 0 and 63. In the present study, a score of <21 points was classified as mild anxiety, 22-35 points as moderate anxiety, and >36 points as severe anxiety. The FSS consists of nine questions, each scoring 0-7 points. The FSS score was calculated by dividing the total score by 9. In the present study, an FSS score of <2.8 was considered no fatigue and >6.1 chronic fatigue syndrome.

The study protocol was approved by the Bursa City Hospital Ethics Committee with the approval number E-13012450-514.99-238900461 and was conducted in accordance with the World Medical Association Declaration of Helsinki. Informed consent was obtained from all patients and healthy controls.

Statistical analysis

IBM SPSS Statistics for Windows, version 25.0 (IBM Corp., Armonk, NY) was used for statistical analysis. Shapiro-Wilk test was used to examine the conformity of the variables to normal distribution. Descriptive statistics were presented using mean ± standard deviation for normally distributed continuous variables, median (25th-75th percentile) for nonnormally distributed continuous variables, and frequency (percentage) for categorical variables. Normally distributed continuous variables were analyzed using the independent samples t-test. Nonnormally distributed continuous variables were analyzed with the Mann-Whitney U test. Categorical variables were analyzed using the chi-square, Fisher's exact, or Fisher-Freeman-Halton tests. Relationships between variables were evaluated with the Pearson or Spearman or point biserial correlation coefficients. p values of less than 0.05 were accepted as statistically significant.

## Results

Characteristics of the patients

The study included 28 patients (20 women and eight men) (18-34 years) and 37 healthy volunteer controls (28 women and nine men) (19-35 years). All patients included in the study were receiving deferasirox chelation therapy. The mean hemoglobin level of the patients was 8.41 ± 0.88 g/dL, and the mean serum ferritin level was 1,328.5 mL/ng (955.0-3,106.5 mL/ng). Eighteen out of the 28 patients (64.29%) had undergone splenectomy. We found no significant differences between groups in terms of age (p = 0.216) or sex (p = 1.000). Table 1 gives a summary of disease-related characteristics.

**Table 1:** Summary of disease-related characteristics of the patients Descriptive statistics were presented by using mean ± SD for normally distributed continuous variables, median (25th-75th percentile) for nonnormally distributed continuous variables, and frequency (percentage) for categorical variables MRI: magnetic resonance imaging; SD: standard deviation

Characteristics	n (%) or mean ± SD
Chelation (15 mg/kg)	
No	0 (0.00%)
Yes	28 (100.00%)
Ferritin (µg/dL)	1,328.5 (955-3,106.5)
Hemoglobin (g/dL; mean)	8.41 ± 0.88
Cardiac MRI (T2)	
No involvement	18 (64.29%)
Mild	3 (10.71%)
Moderate	3 (10.71%)
Severe	4 (14.29%)
Liver MRI (T2)	
No involvement	3 (10.71%)
Mild	8 (28.57%)
Moderate	15 (53.57%)
Severe	2 (7.14%)
Splenectomy	
Absent	10 (35.71%)
Present	18 (64.29%)
Number of transfusions in a year	20.41 ± 4.45

Depression and anxiety assessment results

Thirteen of 28 TM had depression, and 28 TM patients had anxiety symptoms. BDI score (p = 0.003), depression percentage (p = 0.008), and BAI score (p < 0.001) were significantly higher in the patients group than in the control group. We found no significant differences between the groups in terms of anxiety severity (Table 2). The severity of anxiety was predominantly mild in both the controls and patients.

**Table 2:** Summary of depression and anxiety assessment results with regard to groups Descriptive statistics were presented by using median (25th-75th percentile) for nonnormally distributed continuous variables and frequency (percentage) for categorical variables

Depression-anxiety inventory score	Controls (n = 37)	Patients (n = 28)	p
Beck Depression Inventory score	4 (2-8)	11.5 (5-20.5)	0.003
Depression			
No	32 (86.49%)	15 (53.57%)	0.008
Yes	5 (13.51%)	13 (46.43%)	
Beck Anxiety Inventory score	2 (1-4)	13 (4.5-19)	<0.001
Anxiety severity			
Mild	34 (91.89%)	22 (78.57%)	0.185
Moderate	3 (8.11%)	5 (17.86%)	
Severe	0 (0.00%)	1 (3.57%)	

Scores

ISI score (p = 0.027), ISI subthreshold insomnia percentage (p = 0.027), PSQI global score (p = 0.002), PSQI insomnia percentage (p = 0.025), PSQI subjective sleep quality (p = 0.008), PSQI sleep latency (p = 0.003), fatigue score (p = 0.026), and chronic fatigue percentage (p = 0.037) were significantly higher in the patient group than in the controls group. We found no significant differences between the groups in terms of ESS score, ESS classification, PSQI sleep efficiency, and PSQI daytime dysfunction (Table 3).

**Table 3:** Summary of scale scores with regard to groups Descriptive statistics were presented by using mean ± standard deviation for normally distributed continuous variables, median (25th-75th percentile) for nonnormally distributed continuous variables, and frequency (percentage) for categorical variables PSQI: Pittsburgh Sleep Quality Index

Scale scores	Controls (n = 37)	Patients (n = 28)	p
Insomnia Severity Index score	4 (2-8)	9 (2-11)	0.027
Insomnia Severity Index classification			
No clinically significant insomnia	27 (72.97%)	12 (42.86%)	0.027
Subthreshold insomnia	10 (27.03%)	15 (53.57%)	
Moderate insomnia	0 (0.00%)	1 (3.57%)	
Severe insomnia	0 (0.00%)	0 (0.00%)	
Epworth Sleepiness Scale score	3 (2-6)	2 (2-8.5)	0.878
Epworth Sleepiness Scale classification			
Lower normal daytime sleepiness	27 (72.97%)	18 (64.29%)	0.142
Higher normal daytime sleepiness	9 (24.32%)	4 (14.29%)	
Mild excessive daytime sleepiness	1 (2.70%)	3 (10.71%)	
Moderate excessive daytime sleepiness	0 (0.00%)	1 (3.57%)	
Severe excessive daytime sleepiness	0 (0.00%)	2 (7.14%)	
Pittsburgh Sleep Quality Index global score	3.38 ± 1.82	5.57 ± 3.07	0.002
Pittsburgh Sleep Quality Index classification			
Normal	26 (70.27%)	11 (39.29%)	0.025
Insomnia	11 (29.73%)	17 (60.71%)	
PSQI subjective sleep quality	1 (0-1)	1 (1-2)	0.008
PSQI sleep latency	1 (0-1)	1 (1-2)	0.003
PSQI sleep efficiency	0 (0-0)	0 (0-0.5)	0.056
PSQI use of sleep medication	0 (0-0)	0 (0-0)	0.414
PSQI daytime dysfunction	0 (0-1)	0 (0-1)	0.721
Fatigue	2.77 (2.11-4.22)	4.50 (2.05-5.44)	0.026
Fatigue severity			
None	19 (51.35%)	8 (28.57%)	0.037
Mild	18 (48.65%)	17 (60.71%)	
Chronic	0 (0.00%)	3 (10.71%)	

Disease-related variables and scale scores

Ferritin was positively correlated with PSQI subjective sleep quality (r = 0.478, p = 0.010) and fatigue score (r = 0.427, p = 0.023). Heart involvement in magnetic resonance was positively correlated with ISI score (r = 0.426, p=0.024) and ISI classification (r = 0.455, p = 0.015) (Table 4).

**Table 4:** Correlations between disease-related variables and scale scores in the patients group MR: magnetic resonance; PSQI: Pittsburgh Sleep Quality Index; r: correlation coefficient; p: Spearman correlation

Scale scores	p-r values	Ferritin	Hemoglobin	Heart involvement in MR	Liver involvement in MR	Splenectomy	Number of transfusions in a year
Insomnia Severity Index score	r	0.215	0.104	0.426	-0.008	-0.121	-0.186
	p	0.271	0.597	0.024	0.969	0.541	0.353
Insomnia Severity Index classification	r	0.334	0.114	0.455	-0.011	-0.095	-0.322
	p	0.082	0.563	0.015	0.957	0.632	0.101
Epworth Sleepiness Scale score	r	0.320	-0.308	0.312	0.163	-0.179	0.006
	p	0.096	0.110	0.106	0.408	0.363	0.975
Epworth Sleepiness Scale classification	r	0.281	-0.306	0.266	0.092	0.032	-0.082
	p	0.148	0.113	0.171	0.643	0.870	0.684
Pittsburgh Sleep Quality Index global score	r	0.233	0.059	0.334	-0.071	-0.205	-0.060
	p	0.232	0.767	0.082	0.718	0.296	0.766
Pittsburgh Sleep Quality Index classification	r	0.249	-0.054	0.217	0.020	-0.142	-0.039
	p	0.201	0.783	0.267	0.920	0.472	0.847
PSQI subjective sleep quality	r	0.478	-0.187	0.140	0.006	-0.269	0.163
	p	0.010	0.341	0.478	0.975	0.166	0.417
PSQI sleep latency	r	-0.050	0.033	0.167	-0.065	-0.255	0.200
	p	0.801	0.867	0.395	0.743	0.190	0.316
PSQI sleep efficiency	r	0.046	0.289	0.209	-0.070	-0.244	0.014
	p	0.818	0.136	0.287	0.723	0.211	0.943
PSQI use of sleep medication	r	0.145	0.021	0.023	-0.249	0.026	0.110
	p	0.461	0.914	0.906	0.202	0.896	0.584
PSQI daytime dysfunction	r	-0.141	0.108	0.123	0.083	0.021	-0.230
	p	0.474	0.585	0.532	0.673	0.917	0.248
Fatigue	r	0.427	-0.015	0.299	0.176	-0.055	-0.316
	p	0.023	0.940	0.122	0.369	0.780	0.109
Fatigue severity	r	0.325	-0.078	0.065	0.350	-0.106	-0.221
	p	0.092	0.692	0.744	0.067	0.590	0.267

## Discussion

In this study, it was found that depression and anxiety frequency were significantly higher in TM patients compared to healthy controls. Chronic fatigue and insomnia were more common in TM patients, with subjective sleep quality being impaired and sleep latency prolonged. Furthermore, it was observed that individuals with higher ferritin levels had higher scores of subjective sleep quality and fatigue. Additionally, as cardiac involvement increased, the severity of insomnia also increased. The heart's iron buffering systems eventually malfunction. This is more likely to happen if more iron is stored. Increased labile iron levels in the myocyte cause oxidative damage to DNA, iron transporters, and membranes, which leads to arrhythmias, heart failure, and, if left unchecked, ultimately fibrosis. Iron cardiomyopathy is thought to be significantly influenced by the dysregulation of calcium homeostasis, namely the ryanodine channel [22].

Depression and anxiety in TM patients

In previous studies on TM conducted in Turkey, Cakaloz et al. found that the rates of depression and anxiety were 15% and 30% in children with TM, respectively [23]. Yengil et al. reported the rate of depression and anxiety as 20.5% and 38.6%, respectively [24]. In the present study, 46.43% of TM patients had depression, 78.5% had mild anxiety, and 3.57% had severe anxiety. Similar results have been reported by studies conducted in other countries. In the study by Shafiee et al., 62.5% of the patients had depression, according to BDI [25]. The significantly higher anxiety and depression scores observed in the present study compared to the control group are consistent with the literature. The rates of depression and anxiety obtained in the present study are slightly higher compared to the rates previously reported in Turkey. Thus, we believe that this is related to the characteristics of the patient group selected, including age, social factors, and the exclusion of patients using antidepressant and anxiolytic treatments.

Sleep quality and fatigue in TM patients

Sleep disturbances have also been reported in previous studies of anemia types such as iron deficiency anemia and sickle cell anemia. Insomnia, impaired sleep quality, respiratory problems, periodic leg movements during sleep, and restless legs syndrome are commonly observed symptoms in patients with anemia [26,27]. In the literature, high rates of obstructive sleep apnea syndrome (OSAS) have been reported in TM patients [26]. It has been reported that OSAS may be associated with structural changes in the nasopharyngeal area due to extramedullary hematopoiesis (adenoid hypertrophy and lymphoid hyperplasia) [28,29]. In a study by Jahanbakhsh et al., poor sleep quality was observed in 76.2% of TM patients [14]. In the present study, insomnia was noted in 60.71% of TM patients. Compared to the control group, TM patients had higher PSQI global scores, impaired subjective sleep quality, and longer sleep latency. These findings are consistent with the literature. Similar to our findings, previous studies have also identified subjective sleep quality and prolonged sleep latency as the factors most influencing sleep quality [14,30].

Insomnia can affect daytime functioning and lead to increased daytime sleepiness. A PSG study conducted in children found increased daytime sleepiness [26]. In the present study, no difference was found between the patient and control groups regarding daytime sleepiness (ESS). This may be related to the small number of patients and lack of PSG usage. Further studies, including PSG conducted with a larger number of patients, can yield clearer results regarding daytime sleepiness in TM patients.

Fatigue is an important symptom that can affect daily life. Studies on fatigue in TM patients are limited. One of the most common causes of fatigue is anemia [31]. Anemia can cause chronic fatigue, and it is also well-established that fatigue may occur due to transfusions in TM patients [32]. Fatigue has been reported in iron deficiency anemia, sickle cell anemia, and paroxysmal nocturnal hemoglobinuria [33,34]. Anemia reduces tissue oxygenation, diminishes patients' ability to participate in daily activities, and can lead to fatigue, which in turn can result in physiological and emotional burdens, including depression and stress [35]. In the present study, chronic fatigue was observed in 10.71% of TM patients, and the fatigue score was higher than healthy controls. A significant relationship between hemoglobin levels and fatigue was not observed in the patients; however, it was found that as ferritin levels increased, fatigue scores and chronic fatigue increased. This finding raises the question of whether increased transfusion numbers due to severe anemia and transfusion itself may contribute to fatigue. It also suggests that fatigue may have physical and psychological causes. Fatigue can also occur due to tissue iron accumulation resulting from increased blood transfusions, leading to endocrine insufficiency as well as cardiac and hepatic involvement and transmission of hepatitis [36,37].

Laboratory parameters and sleep quality

The ideal ferritin level in thalassemia patients is 1,000-1,500 µg/mL. In the present study, the mean serum ferritin level of the patients was 1,328.5 µg/mL. Organ involvement increases as serum ferritin levels increase. In a previous study conducted in Iran, no relationship was noted between serum ferritin levels and sleep quality, but it was found that individuals with low hemoglobin levels had lower sleep quality [21]. The present study found that PSQI subjective sleep quality increased as ferritin levels increased, and a relationship was found between hemoglobin levels and sleep quality. These findings are inconsistent with the expected results and may be related to the small number of patients.

We were unable to locate any studies in the literature that looked at the connection between TM patients' sleep quality and liver and cardiac MRIs. This is the first study to investigate the correlation between liver and cardiac involvement and sleep quality. The results revealed that ISI scores increased with increasing cardiac involvement, as shown by MRI. No relationship was found between liver involvement in MRI and sleep quality. The increase in insomnia severity with increasing cardiac involvement in MRI could be due to reasons such as heart failure, arrhythmia, and pulmonary hypertension [38]. The present study has certain limitations. First, the study was conducted in a single center, and the number of patients was relatively low. Further multicenter studies with a larger number of patients can yield better and more generalizable results. Furthermore, the data in this study were obtained using subjective tests. More reliable data can be obtained using objective tools such as PSG, and correlating these results with laboratory data can lead to better insights that benefit the patients.

## Conclusions

In conclusion, the decrease in sleep quality in TM patients may be attributed to psychiatric, physical, and laboratory variables. Increased rates of anxiety and depression, stress due to chronic illness, costly treatments, distorted self-image, and expectation of death at an early age affect anxiety and depression, and thus sleep quality. Additionally, abnormal bone structure due to ineffective erythropoiesis can cause sleep apnea syndrome and low hemoglobin levels can cause restless legs syndrome. In addition to organ involvement and laboratory parameters, inquiries should be made about psychological pain, sleep quality, and fatigue during outpatient visits. Patients should be provided with guidance and counseling as well as general health care.
